# Bis[4-amino-3,5-bis­(pyridin-2-yl)-4*H*-1,2,4-triazole-κ^2^ *N* ^2^,*N* ^3^]bis­(benzene-1,2-dicarb­oxy­lic acid-κ*O*)copper(II) bis­(2-carb­oxy­benzoate)

**DOI:** 10.1107/S1600536812000128

**Published:** 2012-01-11

**Authors:** Yan Yan, Wen-Jing Yu, Jing Chen

**Affiliations:** a College of Chemistry, Tianjin Key Laboratory of Structure and Performance for Functional Molecules, Tianjin Normal University, Tianjin 300387, People’s Republic of China

## Abstract

In the complex cation of the title salt, [Cu(C_12_H_10_N_6_)_2_(C_8_H_6_O_4_)_2_](C_8_H_5_O_4_)_2_, the Cu^II^ atom, lying on an inversion center, exhibits a distorted octa­hedral geometry defined by four N atoms from two 4-amino-3,5-bis­(pyridin-2-yl)-4*H*-1,2,4-triazole ligands in the equatorial plane and two axial O atoms from two benzene-1,2-dicarb­oxy­lic acid ligands. In the crystal, the complex cations and the monodeprotonated 2-carb­oxy­benzoate anions are connected by O—H⋯O and N—H⋯O hydrogen bonds, forming a tape along [100]. Adjacent tapes are further linked into a three-dimensional arrangement *via* π–π stacking inter­actions between the triazole and benzene rings and between the pyridine and benzene rings [centroid–centroid distances = 3.6734 (14)/3.9430 (16) and 3.8221 (14) Å]. Intra­molecular N—H⋯N and O—H⋯O hydrogen bonds are also observed.

## Related literature

For the coordination systems of triazole derivatives, see: Chen *et al.* (2011 ▶); Li *et al.* (2010 ▶); Zhang *et al.* (2011 ▶). For the coordination systems of aromatic polycarboxyl­ate ligands, see: Sun *et al.* (2004 ▶); Zehnder *et al.* (2011 ▶). For the coordination systems of mixed ligands, see: Du *et al.* (2005 ▶, 2006 ▶, 2007 ▶, 2008 ▶); Habib *et al.* (2009 ▶).
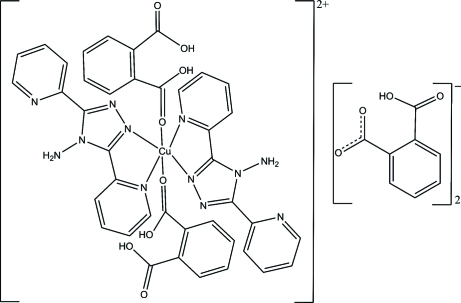


## Experimental

### 

#### Crystal data

[Cu(C_12_H_10_N_6_)_2_(C_8_H_6_O_4_)_2_](C_8_H_5_O_4_)_2_
*M* *_r_* = 1202.56
Monoclinic, 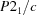

*a* = 12.1171 (7) Å
*b* = 15.9875 (10) Å
*c* = 15.7498 (7) Å
β = 121.739 (3)°
*V* = 2594.8 (2) Å^3^
*Z* = 2
Mo *K*α radiation
μ = 0.51 mm^−1^
*T* = 294 K
0.24 × 0.23 × 0.20 mm

#### Data collection

Bruker APEX CCD diffractometer
Absorption correction: multi-scan (*SADABS*; Sheldrick, 1996 ▶) *T*_min_ = 0.885, *T*_max_ = 0.906
13987 measured reflections
4579 independent reflections
3594 reflections with *I* > 2σ(*I*)
*R*_int_ = 0.023

#### Refinement

*R*[*F* ^2^ > 2σ(*F* ^2^)] = 0.035
*wR*(*F* ^2^) = 0.098
*S* = 1.05
4579 reflections
388 parameters
H-atom parameters constrained
Δρ_max_ = 0.22 e Å^−3^
Δρ_min_ = −0.38 e Å^−3^

### 

Data collection: *SMART* (Bruker, 2007 ▶); cell refinement: *SAINT* (Bruker, 2007 ▶); data reduction: *SAINT*; program(s) used to solve structure: *SHELXS97* (Sheldrick, 2008 ▶); program(s) used to refine structure: *SHELXL97* (Sheldrick, 2008 ▶); molecular graphics: *DIAMOND* (Brandenburg, 1999 ▶); software used to prepare material for publication: *SHELXTL* (Sheldrick, 2008 ▶).

## Supplementary Material

Crystal structure: contains datablock(s) I, global. DOI: 10.1107/S1600536812000128/hy2503sup1.cif

Structure factors: contains datablock(s) I. DOI: 10.1107/S1600536812000128/hy2503Isup2.hkl

Additional supplementary materials:  crystallographic information; 3D view; checkCIF report

## Figures and Tables

**Table 1:** Hydrogen-bond geometry (Å, °)

*D*—H⋯*A*	*D*—H	H⋯*A*	*D*⋯*A*	*D*—H⋯*A*
N5—H5*A*⋯O6	0.90	2.23	2.957 (3)	137
N5—H5*B*⋯N6	0.90	2.23	2.871 (3)	128
O2—H2⋯O8^i^	0.82	1.83	2.619 (2)	161
O3—H3⋯O5^ii^	0.82	1.77	2.579 (2)	171
O7—H7⋯O6	0.82	1.60	2.394 (2)	163

Symmetry codes: (i) 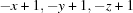; (ii) 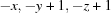.

**Fig. 1.**
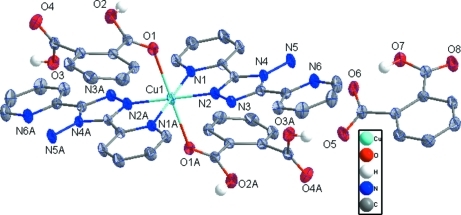
A view of the asymmetric unit of the title compound. Displacement ellipsoids are drawn at the 30% probability level. Except for the carboxyl H atoms, all H atoms were omitted for clarity. [Symmetry code: (A) -x, 1-y, 1-z.]

**Fig. 2.**
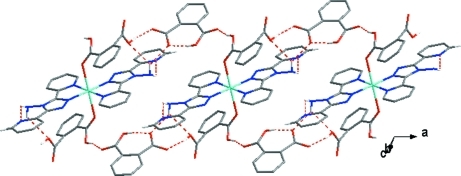
A view of the one-dimensional tape connected via N—H···O and O—H···O hydrogen bonds (red dashed lines).

**Fig. 3.**
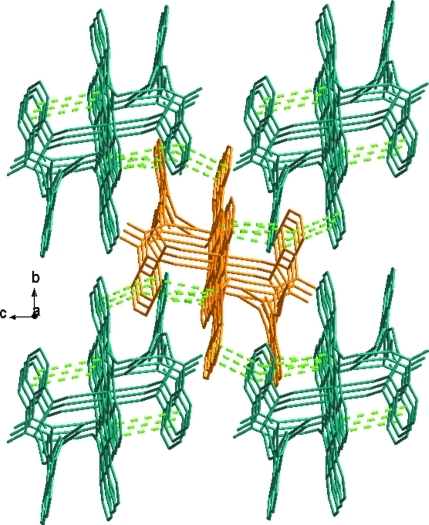
A view of the three-dimensional supramolecular structure constructed via aromatic stacking interactions (green dashed lines).

## supplementary crystallographic information

### Comment

Recently, the derivatives of 1,2,4-triazole have been widely used to synthesize diverse complicated complexes (Chen *et al.*, 2011; Li *et al.*, 2010; Zhang *et al.*, 2011). In addition, the aromatic polycarboxylate ligands can also be regarded as excellent candidates for building coordination frameworks (Sun *et al.*, 2004; Zehnder *et al.*, 2011). With regard to this, the employment of mixed ligands using the derivatives of 1,2,4-triazole and polycarboxylate ligands can be effective in constructing supramolecular structures (Du *et al.*, 2005, 2006, 2007, 2008; Habib *et al.*, 2009). Herein, we present the title complex prepared by the reaction of copper(II) sulfate with benzene-1,2-dicarboxylic acid (H_2_pa) and 4-amino-3,5-bis(pyridin-2-yl)-1,2,4-triazole (2-bpt) as the mixed ligands.

The molecular structure of the title complex is illustrated in Fig. 1. In the [Cu(2-bpt)_2_(H_2_pa)_2_]^2+^ cation, the Cu^II^ atom, lying on an inversion center, shows a distorted octahedral coordination environment defined by four N atoms from two 2-bpt ligands and two O atoms of the carboxylic groups from two H_2_pa ligands. The 2-bpt ligand coordinates to the Cu atom in a bidentate chelating coordination mode, with the *trans*-conformation considering the opposite disposition of two pyridyl N atoms. With regard to the H_2_pa ligand, one carboxylic group adopts a monodentate coordination mode and the other is uncoordinated. As a result, there exists a monodeprotonated Hpa anion in the asymmetric unit to balance the charge of the complex cation.

As shown in Fig. 2, the [Cu(2-bpt)_2_(H_2_pa)_2_]^2+^ cations and the Hpa anions are interconnected to a one-dimensional tape *via* intermolecular O3—H3···O5^i^ and O2—H2···O8^ii^ hydrogen bonds between the carboxyl groups from H_2_pa and Hpa (Table 1) [symmetry codes: (i) -x, 1-y, 1-z; (ii) 1-x, 1-y, 1-z]. The amino group from 2-bpt is involved in an intermolecular N5—H5A···O6 hydrogen bond and an intramolecular N5—H5B···N6 hydrogen bond, which further reinforce the one-dimensional tape. A strong intramolecular O7—H7···O6 hydrogen bond is also observed within the Hpa anion. Furthermore, the adjacent one-dimensional arrays are further extended to afford a three-dimensional supramolecular architecture *via* multiple π–π stacking interactions (Fig. 3). The centroid–centroid distances and the dihedral angles are 3.6734 (14) Å and 3.38 (9)° between the triazole (N2, N3, N4, C6, C7) and benzene (C21^iii^—C26^iii^) rings and 3.8221 (14) Å and 21.59 (7)° between the pyridine (N1, C1–C5) and benzene (C13^i^–C18^i^) rings and 3.9430 (16) Å and 22.25 (8)° between the pyridine (N6, C8–C12) and benzene (C13^iv^–C18^iv^) rings, respectively [symmetry codes: (iii) x, 1/2-y, 1/2+z; (iv) x, 1/2-y, -1/2+z].

### Experimental

A mixture of 2-bpt (23.8 mg, 0.1 mmol), H_2_pa (8.3 mg, 0.05 mmol) and CuSO_4_.5H_2_O (24.9 mg, 0.1 mmol) in water (10 ml) was sealed in a Teflon-lined stainless steel vessel (20 ml), which was heated to 100°C in 24 h and then gradually cooled to room temperature at a rate of 5°C h^-1^. Block blue crystals suitable for X-ray analysis were obtained in 50% yield. Analysis, calculated for C_56_H_42_CuN_12_O_16_: C 55.93, H 3.52, N 13.98%; found: C 55.07, H 3.46, N 13.79%.

### Refinement

All H atoms were initially located in a difference Fourier map and then refined as riding atoms, with C—H = 0.93 (aromatic), N—H = 0.90 (NH_2_) and O—H = 0.82 (OH) Å and with *U*_iso_(H) = 1.2*U*_eq_(C) or 1.5*U*_eq_(N, O).

### Crystal data

**Table:**

[Cu(C_12_H_10_N_6_)_2_(C_8_H_6_O_4_)_2_](C_8_H_5_O_4_)_2_	*F*(000) = 1238
*M**_r_* = 1202.56	*D*_x_ = 1.539 Mg m^−^^3^
Monoclinic, *P*2_1_/*c*	Mo *K*α radiation, λ = 0.71073 Å
Hall symbol: -P 2ybc	Cell parameters from 4780 reflections
*a* = 12.1171 (7) Å	θ = 2.2–25.4°
*b* = 15.9875 (10) Å	µ = 0.51 mm^−^^1^
*c* = 15.7498 (7) Å	*T* = 294 K
β = 121.739 (3)°	Block, blue
*V* = 2594.8 (2) Å^3^	0.24 × 0.23 × 0.20 mm
*Z* = 2	

### Data collection

**Table:**

Bruker APEX CCD diffractometer	4579 independent reflections
Radiation source: fine-focus sealed tube	3594 reflections with *I* > 2σ(*I*)
graphite	*R*_int_ = 0.023
φ and ω scans	θ_max_ = 25.0°, θ_min_ = 2.0°
Absorption correction: multi-scan (*SADABS*; Sheldrick, 1996)	*h* = −14→9
*T*_min_ = 0.885, *T*_max_ = 0.906	*k* = −18→19
13987 measured reflections	*l* = −18→18

### Refinement

**Table:**

Refinement on *F*^2^	Primary atom site location: structure-invariant direct methods
Least-squares matrix: full	Secondary atom site location: difference Fourier map
*R*[*F*^2^ > 2σ(*F*^2^)] = 0.035	Hydrogen site location: inferred from neighbouring sites
*wR*(*F*^2^) = 0.098	H-atom parameters constrained
*S* = 1.05	*w* = 1/[σ^2^(*F*_o_^2^) + (0.051*P*)^2^ + 0.7095*P*] where *P* = (*F*_o_^2^ + 2*F*_c_^2^)/3
4579 reflections	(Δ/σ)_max_ = 0.001
388 parameters	Δρ_max_ = 0.22 e Å^−^^3^
0 restraints	Δρ_min_ = −0.38 e Å^−^^3^

### Special details

**Table:**

Geometry. All e.s.d.'s (except the e.s.d. in the dihedral angle between two l.s. planes) are estimated using the full covariance matrix. The cell e.s.d.'s are taken into account individually in the estimation of e.s.d.'s in distances, angles and torsion angles; correlations between e.s.d.'s in cell parameters are only used when they are defined by crystal symmetry. An approximate (isotropic) treatment of cell e.s.d.'s is used for estimating e.s.d.'s involving l.s. planes.
Refinement. Refinement of *F*^2^ against ALL reflections. The weighted *R*-factor *wR* and goodness of fit *S* are based on *F*^2^, conventional *R*-factors *R* are based on *F*, with *F* set to zero for negative *F*^2^. The threshold expression of *F*^2^ > σ(*F*^2^) is used only for calculating *R*-factors(gt) *etc*. and is not relevant to the choice of reflections for refinement. *R*-factors based on *F*^2^ are statistically about twice as large as those based on *F*, and *R*- factors based on ALL data will be even larger.

### Fractional atomic coordinates and isotropic or equivalent isotropic displacement parameters (Å^2^)

**Table:**

	*x*	*y*	*z*	*U*_iso_*/*U*_eq_	
Cu1	0.0000	0.5000	0.5000	0.03702 (13)	
O5	0.25819 (17)	0.25012 (10)	0.25335 (15)	0.0578 (5)	
O6	0.42188 (18)	0.33760 (10)	0.30636 (14)	0.0557 (5)	
O7	0.61160 (19)	0.35032 (10)	0.29636 (15)	0.0582 (5)	
H7	0.5561	0.3418	0.3103	0.087*	
O8	0.71299 (18)	0.27995 (10)	0.23886 (15)	0.0612 (5)	
N1	0.11634 (16)	0.58006 (11)	0.48080 (13)	0.0357 (4)	
N2	0.12225 (17)	0.41813 (11)	0.49978 (14)	0.0384 (4)	
N3	0.14291 (17)	0.33373 (11)	0.51187 (14)	0.0398 (4)	
N4	0.28569 (17)	0.39093 (11)	0.48400 (13)	0.0364 (4)	
N5	0.38715 (19)	0.40434 (13)	0.46593 (16)	0.0509 (5)	
H5A	0.3530	0.3915	0.4012	0.076*	
H5B	0.4406	0.3643	0.5075	0.076*	
N6	0.4013 (2)	0.22776 (13)	0.50357 (16)	0.0519 (5)	
C1	0.2106 (2)	0.54244 (14)	0.47258 (16)	0.0358 (5)	
C2	0.1071 (2)	0.66321 (14)	0.47397 (18)	0.0430 (5)	
H2A	0.0436	0.6896	0.4805	0.052*	
C3	0.1889 (2)	0.71151 (15)	0.45752 (19)	0.0500 (6)	
H3A	0.1800	0.7694	0.4528	0.060*	
C4	0.2829 (2)	0.67294 (15)	0.4483 (2)	0.0511 (6)	
H4	0.3383	0.7044	0.4368	0.061*	
C5	0.2950 (2)	0.58683 (15)	0.45617 (18)	0.0451 (6)	
H5	0.3587	0.5596	0.4505	0.054*	
C6	0.2094 (2)	0.45237 (13)	0.48423 (15)	0.0346 (5)	
C7	0.2428 (2)	0.31780 (14)	0.50288 (16)	0.0374 (5)	
C8	0.3001 (2)	0.23437 (14)	0.51511 (16)	0.0398 (5)	
C9	0.4507 (3)	0.15095 (18)	0.5139 (2)	0.0652 (8)	
H9	0.5203	0.1444	0.5051	0.078*	
C10	0.4054 (3)	0.08162 (18)	0.5366 (2)	0.0683 (8)	
H10	0.4430	0.0296	0.5426	0.082*	
C11	0.3039 (3)	0.09019 (17)	0.5503 (2)	0.0629 (7)	
H11	0.2719	0.0440	0.5665	0.075*	
C12	0.2495 (2)	0.16788 (15)	0.53986 (18)	0.0488 (6)	
H12	0.1806	0.1755	0.5492	0.059*	
C21	0.4445 (2)	0.19723 (13)	0.26040 (16)	0.0360 (5)	
C22	0.5573 (2)	0.20464 (13)	0.25486 (16)	0.0365 (5)	
C23	0.6072 (2)	0.13201 (14)	0.23817 (19)	0.0465 (6)	
H23	0.6802	0.1362	0.2332	0.056*	
C24	0.5527 (3)	0.05483 (15)	0.2289 (2)	0.0536 (7)	
H24	0.5893	0.0076	0.2188	0.064*	
C25	0.4435 (2)	0.04748 (14)	0.2345 (2)	0.0526 (7)	
H25	0.4056	−0.0046	0.2282	0.063*	
C26	0.3915 (2)	0.11756 (14)	0.24943 (19)	0.0461 (6)	
H26	0.3172	0.1119	0.2525	0.055*	
C27	0.3693 (2)	0.26633 (14)	0.27388 (17)	0.0420 (5)	
C28	0.6319 (2)	0.28304 (14)	0.26291 (18)	0.0423 (5)	
O1	0.14046 (17)	0.52179 (11)	0.68003 (12)	0.0519 (4)	
O2	0.15757 (19)	0.60865 (11)	0.79643 (15)	0.0611 (5)	
H2	0.2051	0.6341	0.7829	0.092*	
O3	−0.12352 (19)	0.62542 (11)	0.74566 (13)	0.0596 (5)	
H3	−0.1594	0.6665	0.7517	0.089*	
O4	−0.0100 (2)	0.62734 (12)	0.91204 (14)	0.0656 (5)	
C13	0.0569 (2)	0.47914 (14)	0.78016 (16)	0.0375 (5)	
C14	0.0682 (2)	0.39419 (15)	0.76748 (17)	0.0450 (6)	
H14	0.1108	0.3770	0.7356	0.054*	
C15	0.0174 (2)	0.33481 (15)	0.8014 (2)	0.0543 (7)	
H15	0.0240	0.2782	0.7910	0.065*	
C16	−0.0429 (3)	0.35987 (16)	0.8505 (2)	0.0555 (7)	
H16	−0.0748	0.3201	0.8752	0.067*	
C17	−0.0561 (2)	0.44334 (16)	0.86325 (18)	0.0502 (6)	
H17A	−0.0970	0.4595	0.8967	0.060*	
C18	−0.0091 (2)	0.50426 (14)	0.82691 (17)	0.0391 (5)	
C19	0.1203 (2)	0.53840 (15)	0.74604 (17)	0.0407 (5)	
C20	−0.0436 (2)	0.59263 (15)	0.83447 (18)	0.0436 (6)	

### Atomic displacement parameters (Å^2^)

**Table:**

	*U*^11^	*U*^22^	*U*^33^	*U*^12^	*U*^13^	*U*^23^
Cu1	0.0356 (2)	0.0354 (2)	0.0541 (2)	0.00117 (16)	0.03318 (19)	0.00233 (17)
O5	0.0499 (11)	0.0419 (10)	0.0929 (13)	0.0050 (8)	0.0454 (10)	0.0014 (9)
O6	0.0716 (12)	0.0328 (9)	0.0875 (13)	−0.0053 (8)	0.0589 (11)	−0.0126 (9)
O7	0.0693 (12)	0.0329 (9)	0.0952 (14)	−0.0116 (8)	0.0589 (12)	−0.0105 (9)
O8	0.0668 (12)	0.0412 (10)	0.1046 (15)	−0.0081 (9)	0.0650 (12)	−0.0038 (9)
N1	0.0327 (10)	0.0386 (11)	0.0434 (10)	0.0011 (8)	0.0253 (9)	0.0018 (8)
N2	0.0353 (10)	0.0378 (10)	0.0512 (11)	0.0023 (8)	0.0290 (9)	0.0039 (8)
N3	0.0385 (10)	0.0367 (10)	0.0531 (11)	0.0022 (8)	0.0302 (10)	0.0019 (9)
N4	0.0326 (10)	0.0420 (11)	0.0438 (10)	0.0018 (8)	0.0266 (9)	−0.0004 (8)
N5	0.0474 (12)	0.0530 (12)	0.0759 (14)	−0.0009 (10)	0.0488 (12)	0.0003 (10)
N6	0.0527 (13)	0.0478 (12)	0.0694 (14)	0.0089 (10)	0.0420 (12)	0.0006 (10)
C1	0.0326 (12)	0.0397 (13)	0.0394 (12)	0.0002 (10)	0.0217 (10)	0.0010 (10)
C2	0.0412 (13)	0.0392 (13)	0.0590 (15)	0.0013 (10)	0.0334 (12)	0.0022 (11)
C3	0.0500 (15)	0.0397 (13)	0.0692 (16)	0.0008 (11)	0.0374 (14)	0.0058 (12)
C4	0.0476 (14)	0.0478 (15)	0.0722 (17)	−0.0042 (12)	0.0412 (14)	0.0073 (13)
C5	0.0406 (13)	0.0450 (14)	0.0670 (16)	0.0012 (11)	0.0400 (13)	0.0047 (12)
C6	0.0295 (11)	0.0416 (13)	0.0396 (12)	−0.0012 (10)	0.0228 (10)	−0.0013 (10)
C7	0.0360 (12)	0.0396 (12)	0.0419 (12)	0.0001 (10)	0.0242 (11)	−0.0011 (10)
C8	0.0376 (12)	0.0438 (13)	0.0396 (12)	0.0040 (10)	0.0215 (11)	−0.0015 (10)
C9	0.0694 (19)	0.0571 (18)	0.088 (2)	0.0191 (15)	0.0542 (18)	0.0044 (15)
C10	0.082 (2)	0.0498 (17)	0.087 (2)	0.0219 (15)	0.0544 (19)	0.0071 (15)
C11	0.079 (2)	0.0455 (15)	0.0756 (19)	0.0079 (14)	0.0483 (17)	0.0111 (13)
C12	0.0495 (15)	0.0489 (15)	0.0549 (15)	0.0031 (12)	0.0321 (13)	0.0028 (12)
C21	0.0402 (12)	0.0276 (11)	0.0426 (12)	0.0021 (9)	0.0234 (11)	0.0029 (9)
C22	0.0380 (12)	0.0288 (11)	0.0436 (12)	−0.0004 (9)	0.0221 (11)	0.0020 (9)
C23	0.0468 (14)	0.0352 (13)	0.0672 (16)	0.0046 (10)	0.0367 (13)	0.0010 (11)
C24	0.0622 (17)	0.0297 (13)	0.0766 (18)	0.0053 (11)	0.0419 (15)	−0.0036 (12)
C25	0.0564 (16)	0.0271 (13)	0.0743 (18)	−0.0044 (11)	0.0343 (14)	−0.0003 (11)
C26	0.0437 (13)	0.0352 (13)	0.0643 (15)	−0.0031 (10)	0.0319 (13)	0.0034 (11)
C27	0.0507 (15)	0.0343 (13)	0.0522 (14)	0.0052 (11)	0.0348 (13)	0.0059 (10)
C28	0.0449 (14)	0.0310 (12)	0.0563 (14)	0.0010 (10)	0.0303 (12)	0.0033 (10)
O1	0.0536 (10)	0.0640 (11)	0.0518 (10)	−0.0052 (8)	0.0372 (9)	−0.0058 (8)
O2	0.0810 (14)	0.0491 (11)	0.0853 (13)	−0.0210 (9)	0.0657 (12)	−0.0156 (10)
O3	0.0661 (12)	0.0499 (11)	0.0610 (11)	0.0186 (9)	0.0321 (10)	0.0014 (9)
O4	0.0877 (14)	0.0598 (12)	0.0613 (12)	0.0002 (10)	0.0474 (11)	−0.0125 (9)
C13	0.0330 (12)	0.0390 (12)	0.0415 (12)	−0.0002 (9)	0.0203 (10)	−0.0006 (10)
C14	0.0412 (13)	0.0446 (14)	0.0500 (14)	0.0053 (11)	0.0245 (12)	−0.0034 (11)
C15	0.0547 (16)	0.0355 (13)	0.0653 (16)	0.0005 (12)	0.0265 (14)	0.0019 (12)
C16	0.0521 (16)	0.0465 (15)	0.0695 (17)	−0.0066 (12)	0.0330 (15)	0.0106 (13)
C17	0.0472 (14)	0.0558 (16)	0.0591 (15)	−0.0019 (12)	0.0358 (13)	0.0038 (12)
C18	0.0361 (12)	0.0405 (13)	0.0437 (12)	−0.0006 (10)	0.0230 (11)	−0.0001 (10)
C19	0.0359 (12)	0.0426 (13)	0.0463 (13)	0.0021 (10)	0.0236 (11)	−0.0014 (11)
C20	0.0437 (14)	0.0451 (13)	0.0544 (15)	−0.0036 (11)	0.0343 (13)	−0.0008 (12)

### Geometric parameters (Å, °)

**Table:**

Cu1—N2	1.9781 (17)	C10—H10	0.9300
Cu1—N1	2.0409 (17)	C11—C12	1.375 (4)
Cu1—O1	2.4455 (17)	C11—H11	0.9300
O5—C27	1.236 (3)	C12—H12	0.9300
O6—C27	1.274 (3)	C21—C26	1.396 (3)
O7—C28	1.277 (3)	C21—C22	1.419 (3)
O7—H7	0.8200	C21—C27	1.516 (3)
O8—C28	1.226 (3)	C22—C23	1.397 (3)
N1—C2	1.334 (3)	C22—C28	1.511 (3)
N1—C1	1.356 (3)	C23—C24	1.371 (3)
N2—C6	1.322 (3)	C23—H23	0.9300
N2—N3	1.367 (3)	C24—C25	1.376 (3)
N3—C7	1.315 (3)	C24—H24	0.9300
N4—C6	1.350 (3)	C25—C26	1.365 (3)
N4—C7	1.374 (3)	C25—H25	0.9300
N4—N5	1.416 (2)	C26—H26	0.9300
N5—H5A	0.9001	O1—C19	1.216 (3)
N5—H5B	0.9014	O2—C19	1.311 (3)
N6—C8	1.334 (3)	O2—H2	0.8200
N6—C9	1.338 (3)	O3—C20	1.321 (3)
C1—C5	1.377 (3)	O3—H3	0.8200
C1—C6	1.453 (3)	O4—C20	1.201 (3)
C2—C3	1.384 (3)	C13—C14	1.390 (3)
C2—H2A	0.9300	C13—C18	1.401 (3)
C3—C4	1.369 (3)	C13—C19	1.487 (3)
C3—H3A	0.9300	C14—C15	1.382 (3)
C4—C5	1.383 (3)	C14—H14	0.9300
C4—H4	0.9300	C15—C16	1.373 (4)
C5—H5	0.9300	C15—H15	0.9300
C7—C8	1.469 (3)	C16—C17	1.371 (4)
C8—C12	1.382 (3)	C16—H16	0.9300
C9—C10	1.365 (4)	C17—C18	1.394 (3)
C9—H9	0.9300	C17—H17A	0.9300
C10—C11	1.362 (4)	C18—C20	1.496 (3)
			
N2—Cu1—N2^i^	180.0	C10—C11—H11	120.4
N2—Cu1—N1^i^	99.29 (7)	C12—C11—H11	120.4
N2^i^—Cu1—N1^i^	80.71 (7)	C11—C12—C8	118.2 (2)
N2—Cu1—N1	80.71 (7)	C11—C12—H12	120.9
N2^i^—Cu1—N1	99.29 (7)	C8—C12—H12	120.9
N1^i^—Cu1—N1	180.00 (8)	C26—C21—C22	117.6 (2)
N2—Cu1—O1	91.79 (7)	C26—C21—C27	114.24 (19)
N2^i^—Cu1—O1	88.21 (7)	C22—C21—C27	128.08 (19)
N1^i^—Cu1—O1	91.79 (6)	C23—C22—C21	117.9 (2)
N1—Cu1—O1	88.21 (6)	C23—C22—C28	113.98 (19)
C28—O7—H7	109.5	C21—C22—C28	128.06 (19)
C2—N1—C1	118.25 (18)	C24—C23—C22	122.4 (2)
C2—N1—Cu1	127.02 (15)	C24—C23—H23	118.8
C1—N1—Cu1	114.72 (14)	C22—C23—H23	118.8
C6—N2—N3	109.19 (17)	C23—C24—C25	119.7 (2)
C6—N2—Cu1	113.48 (14)	C23—C24—H24	120.1
N3—N2—Cu1	137.33 (14)	C25—C24—H24	120.1
C7—N3—N2	106.71 (18)	C26—C25—C24	119.2 (2)
C6—N4—C7	106.33 (17)	C26—C25—H25	120.4
C6—N4—N5	123.92 (18)	C24—C25—H25	120.4
C7—N4—N5	129.74 (18)	C25—C26—C21	123.1 (2)
N4—N5—H5A	105.3	C25—C26—H26	118.5
N4—N5—H5B	96.6	C21—C26—H26	118.5
H5A—N5—H5B	112.8	O5—C27—O6	122.6 (2)
C8—N6—C9	116.1 (2)	O5—C27—C21	117.6 (2)
N1—C1—C5	122.4 (2)	O6—C27—C21	119.8 (2)
N1—C1—C6	111.28 (18)	O8—C28—O7	121.4 (2)
C5—C1—C6	126.3 (2)	O8—C28—C22	118.8 (2)
N1—C2—C3	122.3 (2)	O7—C28—C22	119.8 (2)
N1—C2—H2A	118.9	C19—O1—Cu1	133.81 (16)
C3—C2—H2A	118.9	C19—O2—H2	109.5
C4—C3—C2	119.1 (2)	C20—O3—H3	109.5
C4—C3—H3A	120.5	C14—C13—C18	118.9 (2)
C2—C3—H3A	120.5	C14—C13—C19	117.5 (2)
C3—C4—C5	119.6 (2)	C18—C13—C19	123.6 (2)
C3—C4—H4	120.2	C15—C14—C13	121.2 (2)
C5—C4—H4	120.2	C15—C14—H14	119.4
C1—C5—C4	118.4 (2)	C13—C14—H14	119.4
C1—C5—H5	120.8	C16—C15—C14	119.6 (2)
C4—C5—H5	120.8	C16—C15—H15	120.2
N2—C6—N4	108.30 (18)	C14—C15—H15	120.2
N2—C6—C1	119.71 (18)	C17—C16—C15	120.3 (2)
N4—C6—C1	131.98 (19)	C17—C16—H16	119.9
N3—C7—N4	109.44 (19)	C15—C16—H16	119.9
N3—C7—C8	124.1 (2)	C16—C17—C18	121.0 (2)
N4—C7—C8	126.45 (19)	C16—C17—H17A	119.5
N6—C8—C12	123.6 (2)	C18—C17—H17A	119.5
N6—C8—C7	117.3 (2)	C17—C18—C13	119.0 (2)
C12—C8—C7	119.1 (2)	C17—C18—C20	115.9 (2)
N6—C9—C10	124.1 (3)	C13—C18—C20	125.0 (2)
N6—C9—H9	117.9	O1—C19—O2	123.0 (2)
C10—C9—H9	117.9	O1—C19—C13	123.0 (2)
C11—C10—C9	118.7 (3)	O2—C19—C13	114.0 (2)
C11—C10—H10	120.6	O4—C20—O3	124.1 (2)
C9—C10—H10	120.6	O4—C20—C18	123.9 (2)
C10—C11—C12	119.2 (3)	O3—C20—C18	111.8 (2)
			
N2—Cu1—N1—C2	−178.4 (2)	N6—C9—C10—C11	−0.3 (5)
N2^i^—Cu1—N1—C2	1.6 (2)	C9—C10—C11—C12	0.7 (4)
O1—Cu1—N1—C2	−86.26 (19)	C10—C11—C12—C8	0.4 (4)
N2—Cu1—N1—C1	2.92 (15)	N6—C8—C12—C11	−2.0 (4)
N2^i^—Cu1—N1—C1	−177.08 (15)	C7—C8—C12—C11	179.5 (2)
O1—Cu1—N1—C1	95.02 (15)	C26—C21—C22—C23	0.4 (3)
N1^i^—Cu1—N2—C6	177.47 (15)	C27—C21—C22—C23	−177.5 (2)
N1—Cu1—N2—C6	−2.53 (15)	C26—C21—C22—C28	179.2 (2)
O1—Cu1—N2—C6	−90.42 (15)	C27—C21—C22—C28	1.2 (4)
N1^i^—Cu1—N2—N3	−2.8 (2)	C21—C22—C23—C24	−1.1 (4)
N1—Cu1—N2—N3	177.2 (2)	C28—C22—C23—C24	179.9 (2)
O1—Cu1—N2—N3	89.3 (2)	C22—C23—C24—C25	1.0 (4)
C6—N2—N3—C7	−0.3 (2)	C23—C24—C25—C26	−0.1 (4)
Cu1—N2—N3—C7	179.97 (17)	C24—C25—C26—C21	−0.6 (4)
C2—N1—C1—C5	−0.9 (3)	C22—C21—C26—C25	0.4 (4)
Cu1—N1—C1—C5	177.90 (17)	C27—C21—C26—C25	178.6 (2)
C2—N1—C1—C6	178.53 (19)	C26—C21—C27—O5	−14.8 (3)
Cu1—N1—C1—C6	−2.6 (2)	C22—C21—C27—O5	163.2 (2)
C1—N1—C2—C3	0.9 (3)	C26—C21—C27—O6	164.4 (2)
Cu1—N1—C2—C3	−177.74 (18)	C22—C21—C27—O6	−17.6 (3)
N1—C2—C3—C4	−0.3 (4)	C23—C22—C28—O8	11.6 (3)
C2—C3—C4—C5	−0.4 (4)	C21—C22—C28—O8	−167.2 (2)
N1—C1—C5—C4	0.3 (4)	C23—C22—C28—O7	−166.9 (2)
C6—C1—C5—C4	−179.1 (2)	C21—C22—C28—O7	14.3 (4)
C3—C4—C5—C1	0.4 (4)	N2—Cu1—O1—C19	−148.0 (2)
N3—N2—C6—N4	1.2 (2)	N2^i^—Cu1—O1—C19	32.0 (2)
Cu1—N2—C6—N4	−179.01 (13)	N1^i^—Cu1—O1—C19	−48.7 (2)
N3—N2—C6—C1	−177.86 (18)	N1—Cu1—O1—C19	131.3 (2)
Cu1—N2—C6—C1	1.9 (2)	C18—C13—C14—C15	−0.9 (3)
C7—N4—C6—N2	−1.6 (2)	C19—C13—C14—C15	176.9 (2)
N5—N4—C6—N2	177.72 (18)	C13—C14—C15—C16	−1.5 (4)
C7—N4—C6—C1	177.3 (2)	C14—C15—C16—C17	2.0 (4)
N5—N4—C6—C1	−3.4 (4)	C15—C16—C17—C18	0.1 (4)
N1—C1—C6—N2	0.5 (3)	C16—C17—C18—C13	−2.5 (4)
C5—C1—C6—N2	179.9 (2)	C16—C17—C18—C20	172.8 (2)
N1—C1—C6—N4	−178.3 (2)	C14—C13—C18—C17	2.9 (3)
C5—C1—C6—N4	1.1 (4)	C19—C13—C18—C17	−174.8 (2)
N2—N3—C7—N4	−0.7 (2)	C14—C13—C18—C20	−172.0 (2)
N2—N3—C7—C8	177.3 (2)	C19—C13—C18—C20	10.3 (4)
C6—N4—C7—N3	1.4 (2)	Cu1—O1—C19—O2	−114.9 (2)
N5—N4—C7—N3	−177.8 (2)	Cu1—O1—C19—C13	68.1 (3)
C6—N4—C7—C8	−176.6 (2)	C14—C13—C19—O1	26.3 (3)
N5—N4—C7—C8	4.2 (4)	C18—C13—C19—O1	−155.9 (2)
C9—N6—C8—C12	2.3 (4)	C14—C13—C19—O2	−150.9 (2)
C9—N6—C8—C7	−179.2 (2)	C18—C13—C19—O2	26.9 (3)
N3—C7—C8—N6	179.9 (2)	C17—C18—C20—O4	62.5 (3)
N4—C7—C8—N6	−2.4 (3)	C13—C18—C20—O4	−122.5 (3)
N3—C7—C8—C12	−1.6 (3)	C17—C18—C20—O3	−112.5 (2)
N4—C7—C8—C12	176.1 (2)	C13—C18—C20—O3	62.5 (3)
C8—N6—C9—C10	−1.1 (4)		

Symmetry codes: (i) −*x*, −*y*+1, −*z*+1.

### Hydrogen-bond geometry (Å, °)

**Table:**

*D*—H···*A*	*D*—H	H···*A*	*D*···*A*	*D*—H···*A*
N5—H5A···O6	0.90	2.23	2.957 (3)	137
N5—H5B···N6	0.90	2.23	2.871 (3)	128
O2—H2···O8^ii^	0.82	1.83	2.619 (2)	161
O3—H3···O5^i^	0.82	1.77	2.579 (2)	171
O7—H7···O6	0.82	1.60	2.394 (2)	163

Symmetry codes: (ii) −*x*+1, −*y*+1, −*z*+1; (i) −*x*, −*y*+1, −*z*+1.
